# Anchor Away – A Fast, Reliable and Reversible Technique To Inhibit Proteins in *Drosophila melanogaster*

**DOI:** 10.1534/g3.120.401055

**Published:** 2020-03-26

**Authors:** Pablo Sanchez Bosch, Julia Pepperl, Konrad Basler

**Affiliations:** Institute of Molecular Life Sciences, University of Zürich, Zürich, Switzerland, CH-8057

**Keywords:** Drosophila melanogaster, mTOR, rapamycin, pygopus, brinker

## Abstract

Several techniques have been developed to study specific gene function in loss-of-function situations. In *Drosophila melanogaster*, RNAi and the generation of mutant clones are widely used. However, both techniques have the limitation that there is a significant time lag before gene function is abolished. Given the relatively rapid development of *Drosophila*, such perdurance is a serious impediment to study gene function. Here we describe the adaptation of the anchor-away technique for use in *Drosophila*. Anchor-away was originally developed in yeast to quickly and efficiently abrogate the function of nuclear proteins by sequestering - anchoring - them away in a different cellular compartment. The required components are present in the cells, and the system is triggered by the addition of rapamycin, resulting in a rapid generation of a loss-of-function situation. We provide here proof of principle for the system by producing loss-of-function situations for two nuclear proteins – Pygopus and Brinker. The system allows to study the requirement of any protein during any time window, and at the same time circumvents difficulties, such as off-target effects or variable phenotypes, which are inherent in other techniques, for example RNAi.

Loss-of-function (LOF) experiments have been performed for decades to study gene function. In *Drosophila*, several methods have been developed and extensively used (Cooley *et al.* 1998, Adams & Sekelsky 2002). Mutagenesis screens have led to the discovery of the function of hundreds of proteins and were integral in the quest for identifying pathway components in the embryo (Nüsslein-Volhard & Wieschaus 1980; St Johnston 2002; Jenny & Basler 2014). To facilitate the study of the function of an essential gene at later stages, techniques were developed that interfered with gene function only regionally (induction of genetic mosaics) or only transiently (conditional alleles). The preeminent approach that allowed the generation of mosaic LOF situations in tissues was the generation of mitotic recombinant clones (Xu & Rubin 1993). RNAi (Fire *et al.* 1998) was the most widely adopted and extensively used method to transiently down-regulate gene expression in *Drosophila*; one reason for this was the generation of libraries where almost any gene in the genome could be targeted, allowing scientists to performing reverse genetics. Although powerful, these methods have a major drawback: they do not directly target the protein but act on the gene or the mRNA level and are thus sensitive to issues such as protein half-life, causing a delay before the LOF takes effect in the tissue (Boutros & Ahringer 2008).

To overcome this problem, several approaches have been developed to achieve a more rapid and efficient LOF by targeting the protein directly. These methods rely on targeted protein degradation, cleavage or sequestering (Harmansa *et al.* 2017; Haruki *et al.* 2008; Caussinus *et al.* 2011). One of these methods, developed in yeast, is the anchor-away technique. LOF is achieved by sequestering the target protein in another compartment of the cell where it is unable to perform its physiological function. This sequestering is triggered by the addition of rapamycin, allowing investigators to trigger the LOF at any time point. The effect of the anchor-away method is essentially instantaneous as all the necessary components are already present in the cell, and the loss of function is triggered by the addition of rapamycin (Haruki *et al.* 2008).

The technique is based on a binary system whose components have to be integrated beforehand: an anchor protein (by which the protein of interest will be sequestered) and an engineered target protein. The anchoring process is based on the interaction between the human FK506 binding protein (FKBP12), and the 11 kD FKBP12-rapamycin-binding (FRB) domain of the human mTor (Chen *et al.* 1995; Belshaw *et al.* 1996). Rapamycin binds to FKBP12, and this creates an interaction surface for FRB, which binds and forms a tight ternary complex (Chen *et al.* 1995). By tagging the anchor with FKBP12 and the target with FRB, the two proteins will bind strongly to each other after the addition of rapamycin. As a consequence, the target will be sequestered to the subcellular compartment where the anchor is located (Figure 1A).

**Figure 1:**
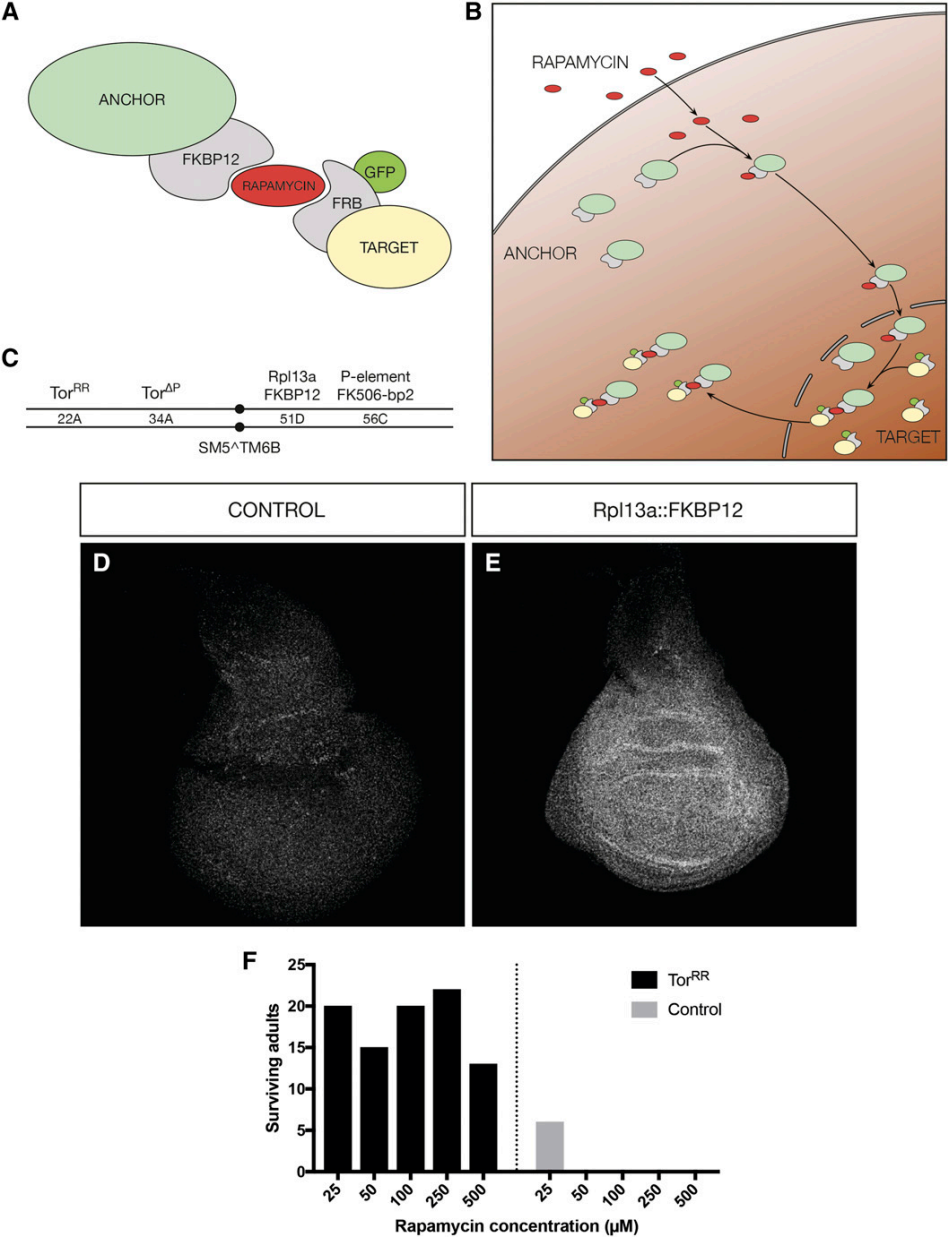
Adapting the anchor-away to *Drosophila*. A) Schematic of the anchor-away components. B) Schematic of the mechanism of action of the anchor-away upon rapamycin addition. The anchor first binds rapamycin, and this complex drives the capture of the target protein to the cytoplasm. C) Chromosomal localization of the anchor-away components in the second chromosome. The components are either homozygous or balanced over SM5^TM6b. D-E) Staining against human FKBP12 in control discs without Rpl13a::FKBP12 (D), or discs from larvae carrying Rpl13a::FKBP12 (E). F) Survival of animals upon different rapamycin treatments with and without Tor^RR^.

There are various possibilities when anchoring proteins away, depending on the subcellular location of the protein of interest. For nuclear proteins, a cytoplasmic anchor is an obvious choice, and ribosomal proteins have been shown to be suitable for this, as once ribosomes are assembled, they will remain cytoplasmic (Haruki *et al.* 2008). In addition, ribosomal proteins translocate to the nucleus after biosynthesis, where they combine with the different rRNA molecules to assemble ribosomes. Afterward, the large and small ribosomal complexes are translocated to the cytoplasm (Zemp & Kutay 2007; Köhler & Hurt 2007). In this process, the target protein will also bind to the ribosomal protein anchor, and is subsequently translocated to the cytoplasm, where it is prevented from going back to the nucleus (Figure 1B). If the target protein is cytoplasmic, a membrane-bound anchor has been shown to be efficient (Tsuchiya *et al.* 2013). The development of a suitable anchor is key to this method and will depend mainly on the cellular localization of the target to be anchored away.

In the present work, we adapted the anchor-away technique to *Drosophila*: we devised a ribosomal protein anchor to be able to study LOF of *Drosophila* nuclear proteins. As a proof of principle, we have tested the technique with two nuclear factors of independent pathways – Pygopus (Wingless signaling) and Brinker (Decapentaplegic signaling). The LOF phenotypes confirmed the specificity and efficiency of this system.

## Material and methods

### Drosophila strains

The following fly stocks were used for the experiments: *Tor*^ΔP^ and *22A-Tor^S1956T^* (Zhang *et al.* 2000), *FK506-bp2* Kyoto stock 205244 (*P{GSV6}GS10737)* (Toba *et al.* 1999), *brk*^M68^ (Jaźwińska *et al.* 1999), *56C-Rpl13a*::*FKBP12*, *86Fb-FRBGFP*::*Brk*, *FRBGFP*::*Pygo*, *C765-Gal4*, Vienna RNAi stocks v19692, v19693, v100724, v2919 and v100824.

### Cloning procedures

*rpl13a* was tagged with *2xFKBP12* under the control of its own promoter, endogenous 5′ and 3′ UTRs and supposedly all its endogenous regulatory regions. The resulting transgene was integrated via the Φ*C31* integrase system into the landing site at 51D (Bischof *et al.* 2007).

*FRB-GFP*::*Brk* was generated by fusing the *FRB-GFP* cassette into a BAC containing the whole *brk* genomic region by BAC recombineering (Warming *et al.* 2005). The resulting vector was integrated via the Φ*C31* integrase into the landing site at *86Fb* (Bischof *et al.* 2007).

For *FRBGFP*::*Pygo*, CRISPR gRNA were cloned in pU6-BbsI-gRNA (Gratz *et al.* 2013). A donor plasmid (pFRBGFP) was generated by using the pDsRed-attP plasmid as a backbone. We replaced the fragment between the multiple cloning sites for the homology regions with an *in frame FRBGFP* DNA fragment, by digesting the plasmid with AarI and SapI and cloning a PCR fragment containing the *FRB-GFP* fragment in such a way that it will be *in frame* once the homology arms are cloned in the plasmid. gRNA and donor plasmid were co-injected into embryos expressing *nos-Cas9* (Port *et al.* 2014). The F1 was screened by PCR to confirm the insertion of the FRBGFP fragment in the correct region.

### Immunostaining

Third instar imaginal discs were dissected in PBS and fixed during 30 min with 4% Formaldehyde in PBS. Prior to antibody staining, discs were blocked with 2% heat inactivated goat serum (HINGS) and stained overnight with primary antibodies. The following antibodies and concentrations were used: guinea pig α-Sens (Nolo *et al.* 2000), 1:1000; guinea pig α-Brk (Doumpas *et al.* 2013), 1:500; Cell Signaling mouse α-FKBP12, 1:500. Secondary antibody staining was performed for 2 hr, using Thermo Fisher Alexa antibodies. Discs were mounted in Vectashield and images were taken with a Zeiss LSM710 confocal microscope.

### Rapamycin culture ex vivo

Imaginal wing discs were dissected in Wing Medium 1 (WM1) (Restrepo *et al.* 2016) and transferred to reaction tubes. The solution was replaced by WM1 containing rapamycin 50 µM and incubated for 1 to 4 hr. After incubation, Rapamycin was removed and discs were fixed and stained as described in the prior section.

### Data availability

All plasmids, fly strains and reagents used for the study are available upon request. The authors affirm that all data necessary for confirming the conclusions of the article are present within the article, figures, and tables. Supplemental material available at figshare: https://doi.org/10.25387/g3.11959209.

## Results

### Adapting the anchor-away system to Drosophila

To test and apply the anchor-away method in *Drosophila melanogaster* it was necessary to generate an anchor appropriate for a functional target. In addition, the modification of various genes was required (Figure 1C). First, we wanted to generate a genetic background in which *Drosophila* is insensitive to rapamycin. The primary target of rapamycin is *Tor*. We thus introduced a *tor* transgene with the mutation S1956T. This mutation renders *Tor* rapamycin resistant (Zhang *et al.* 2000). It was crossed into a *tor* null mutant background (*tor^∆P^*).

Second, to avoid potential competitive binding to rapamycin, we also abolished the expression of an endogenous FK506 binding protein, the *Drosophila* homolog of the yeast FPR1, *FK506-bp2*. For this we used a null allele, which carried a *P*-element insertion in the second exon of *FK506-bp2* (Toba *et al.* 1999).

Next, we generated a protein anchor, which we wanted to be expressed ubiquitously and at high levels to ensure efficient sequestering of the target. We selected the ribosomal protein Rpl13a, the homolog of the protein used in the yeast system (Haruki *et al.* 2008). This protein has an exposed C-terminus (Haruki *et al.* 2008, Anger *et al.* 2013), allowing it to be fused to two copies of the human FKBP12 rapamycin-binding domain. These modifications were made in the context of a genomic rescue construct such that the gene was controlled by its endogenous regulatory elements. The construct was integrated in the second chromosome via *attB/attP* integration (Bischof *et al.* 2007), and proper localization of the protein was assessed by immunostaining (Figure 1D-E). In parallel, we generated and introduced a transgene, *UAS-FKBP12*::*Rpl13a*, that could potentially be used to restrict the anchoring to a subset of cells by using compartment- or tissue-specific Gal4 lines.

Once all transgenes were integrated, we assessed if the animals were viable when exposed to rapamycin. We transferred eggs into food containing rapamycin, from 20 to 500 µM to determine a concentration that will affect WT but not the rapamycin-resistant anchor-away larvae. Based on the viability, we concluded that a concentration of 50 µM rapamycin was optimal (Figure 1F), similar to what has been used in prior reports in which *Tor* signaling was studied in *Drosophila* (Oldham *et al.* 2000). Lower amounts of rapamycin allowed some WT larvae to develop, and very high concentrations affected even the transgenic anchor-away strain.

### Generation of anchorable Brinker and Pygopus variants

To test the feasibility of anchoring nuclear proteins in *Drosophila*, we generated FRB-tagged variants of two well-studied nuclear factors — Brinker (Brk) and Pygopus (Pygo). Brk is the main effector of the Decapentaplegic (Dpp) pathway in *Drosophila*. Its expression domain in the wing imaginal disc is restricted to the lateral regions, and a *brk* LOF produces a clear overgrowth phenotype and derepression of Dpp target genes (Jaźwińska *et al.* 1999). Pygo is one the binding partners of Armadillo, the fly homolog of β-catenin. Its recruitment is critical in the signal transduction of Wingless (Wg) target genes (Kramps *et al.* 2002). Mutants for *pygo* present severe undergrowth phenotypes. In addition to the rapamycin binding FRB tag, we also added the *eGFP* sequence (FRB-GFP), to be able to directly localize the anchored targets. The targets were engineered by using two different approaches.

**Figure 2:**
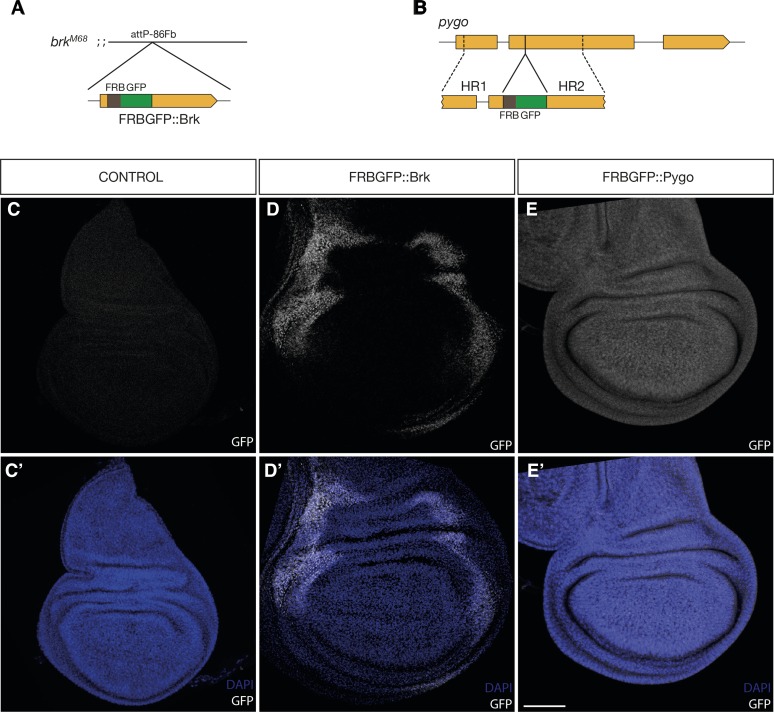
Establishing anchorable Brk and Pygo. A) Schematic of the *brk* BAC rescue. This BAC was integrated on the third chromosome and coupled with the null allele brkM68. B) Schematic of the modification of the *pygo* locus by CRISPR/Cas9. FRBGFP was integrated in frame right after the 5′UTR in the second exon of *pygo*. C-E’) Detection of the GFP-tagged targets. C-C’) Control discs without anchor-away target. D-D’) Discs carrying FRBGFP::Brk over a *brk*-null background. The shape of the disc is normal and Brk is localized in the normal expressing region and is present in the nucleus. E-E’) Discs carrying homozygous FRBGFP::Pygo. Discs retain a WT-like shape and Pygo is produced ubiquitously and localizes in the nucleus as expected. Scalebar = 50 μM.

**Figure 3:**
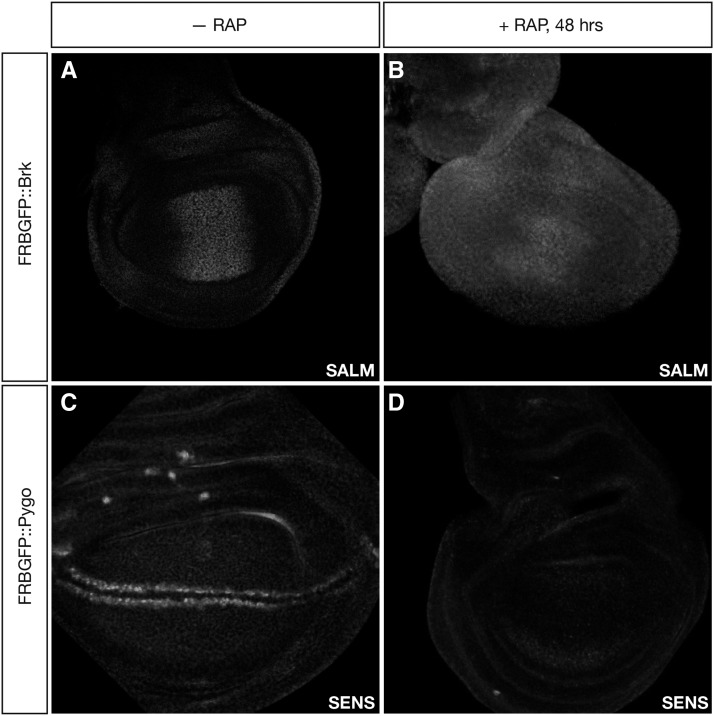
Pygo and Brk are successfully anchored *in vivo*. A) FRBGFP::Brk disc without rapamycin treatment. B) FRBGFP::Brk disc dissected 48 hr after feeding rapamycin to larvae. C) Control disc carrying FRBGFP::Pygo, without rapamycin treatment. D) Disc carrying FRBGFP::Pygo dissected 48 hr after feeding rapamycin to larvae.

To generate *FRB-GFP*::*brk*, we introduced the *FRB-GFP* tag in the N-terminal end of *brk*, and cloned it in a BAC construct to integrate the whole genomic fragment via *attB/attP* in the third chromosome, in the *attP-86Fb* locus (Bischof *et al.* 2007), depicted in Figure 2A. Both the expression pattern and the subcellular localization of the fusion protein, assessed by GFP expression, were the same as those of the endogenous Brk protein (Figure 2D-D’). To assess the efficiency of the anchoring, the transgenic construct was crossed into a *brk*^M68^* null* background (Jaźwińska *et al.* 1999), which also contained the other anchor-away transgenes. Anchoring of FRB-GFP::Brk by rapamycin exposure was lethal at the third larval stage. We assessed the effect of Brk sequestration by observing the phenotypes of third instar larval wing discs. Discs from rapamycin-treated animals exhibited overgrowth, resembling the phenotype of *brk* LOF. The effect in Dpp signaling was confirmed by immunostaining against the downstream target *spalt major* (*salm*). Discs where Brk was anchored away showed widespread derepression of Salm, as it is expected for a *brk* LOF situation (Figure 3A-B).

To generate a FRB-GFP-tagged Pygo, we used the CRISPR/Cas9 method (Port *et al.* 2014). We recombined the *FRB-GFP* fragment in frame at the beginning of the open reading frame of the endogenous *pygo* gene (Figure 2B). As with Brk, Pygo expression was unaffected by the modification and showed the expected localization (Figure 2E-E’). Homozygous *FRB-GFP*::*pygo* animals carrying the anchor-away transgenes were exposed to rapamycin. This caused a developmental arrest, and larvae were not able to pupariate. To determine the functional consequence of anchoring-away Pygo, we assayed the expression of the Wg target gene *Senseless* (*Sens*) by immunostaining. In larvae fed with rapamycin for 48 hr, Sens expression was completely abolished, confirming the inactivation of the Wg pathway (Figure 3C-D).

These results demonstrate that the Anchor-away method works in *Drosophila* to induce an acute LOF situation.

### Anchoring away as a fast and efficient knock-down system

We next wanted to assess how rapidly the anchor-away method creates a LOF situation. Due to the ubiquitous localization of Pygo in the wing disc, we used FRB-GFP::Pygo to measure how fast the cytoplasmic anchor traps a nuclear target in *Drosophila*.

**Figure 4:**
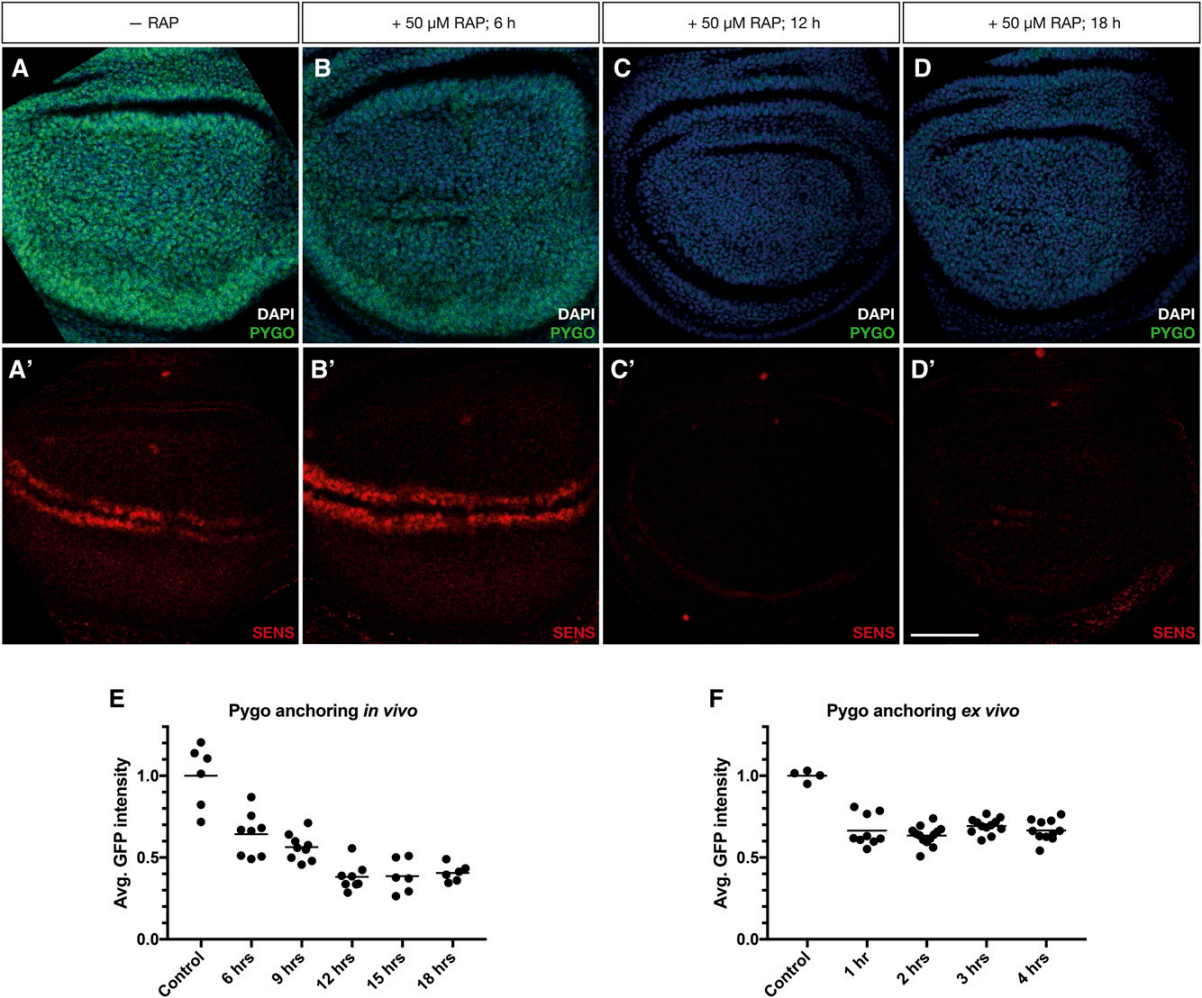
Pygo anchoring *in vivo* achieves a LOF phenotype in 12 hr. A-D) Pygo (green) fluorescence decays over time after rapamycin treatment. A’-D’) Sens is undetectable 12 hr after rapamycin treatment. A, A’) Control discs without rapamycin treatment, B-D’) Representative discs from larvae fed with rapamycin for 6 hr (B, B’), 12 hr (C, C’) and 18 hr (D, D’). E) Decay in GFP::Pygo fluorescence after feeding larvae with rapamycin. The minimal intensity is achieved already at 6 hr after rapamycin treatment. F) Decay in GFP::Pygo fluorescence after treating dissected discs with 50 mM rapamycin. The maximal decay happens in the timeframe of 1 hr and is maintained thereafter. Scalebar = 50 µM.

We first assessed how quickly after feeding larvae with rapamycin relocalization of FRB-GFP::Pygo occurs. We collected eggs in regular food and let the ensuing larvae develop until third instar. We then transferred them to rapamycin-containing food and dissected them at defined time points. To determine when the effect of Pygo anchoring away affected target genes, we again used Sens protein levels as a readout (Figure 4A’-D’). We found that 12 hr after treatment Sens protein was not detectable anymore (Figure 4C’). Pygo localization was also affected (Figure 4A-D, E). As early as 6 hr after rapamycin feeding, there is a clear decay in the nuclear FRB-GFP::Pygo signal (Figure 4B, E). At 12 hr after treatment, the Pygo signal was much lower than the control without treatment (Figure 4C,E). We hypothesize that the decay in signal is due to the sequestering of Pygo to the cytoplasm. As the protein is now more diffuse, the fluorescent signal is also more delocalized. We measured the amount of Pygo by Western blot to find out if cytoplasmically anchored Pygo is degraded at a higher rate by the proteasome, causing the decrease of the signal. However, Pygo levels did not change over the course of 18 hr (Suppl. Figure 1), in contrast with the decay in fluorescence signal (≥ 50%).

In conclusion, the effect of the anchor-away *in vivo* is detectable as early as 6 hr after rapamycin feeding, and the pathway is inhibited 12 hr after treatment.

To assess how rapidly Pygo is anchored *ex vivo*, we dissected third instar discs and applied rapamycin-containing Wing Medium 1 (Restrepo *et al.* 2016). We then measured the anchoring efficiency *ex vivo* by the fluorescence decay due to protein diffusion to the cytoplasm, as it was the fastest readout of the rapamycin-induced anchoring *in vivo* (Figure 4E). When cultured in rapamycin, most nuclear Pygo was anchored already 1 hr after exposure to rapamycin, and Pygo signal was low for at least 4 hr of culture (Figure 3H). Therefore, anchoring proteins away is also feasible and highly efficient in *Drosophila* cultured discs.

### Depletion via anchor-away is more effective than RNAi downregulation

RNAi-mediated downregulation (Fire *et al.* 1998) is widely used in *Drosophila*, in part due to the existence of collections targeting all the genes in the genome. Other advantage is the possibility of spatio-temporal control of the knock-down (Dietzl *et al.* 2007; Ni *et al.* 2009). Despite these benefits, RNAi downregulation has drawbacks, such as off-target effects, that can confound analyses and high variability or delayed repression of targets (Boutros & Ahringer 2008).

The anchor-away technique is a viable alternative to RNAi, especially for studies of a small number of genes. One of the major impediments of RNAi is that its efficiency can vary greatly (Boutros & Ahringer 2008). We tested two different RNAi lines which target *pygo* and two RNAi lines targeting *brk* and compared the efficiency of their knock-down functionally examining the effect on Sens and Salm levels, respectively (Suppl. Figure 2). We triggered expression of the RNAi transgene for 48 hr by using the disc driver *C765-Gal4*. Although both RNAi targeting *pygo* were able to decrease its levels up to the point where Sens was barely detectable (Suppl. Figure 2A-C), in some discs Sens levels were still high, or not affected at all (Suppl. Figure 2B’-C’). In addition, the RNAi constructs against *brk* were not able to decrease Brk function significantly when driven by C765-Gal4, as *salm* expression was unaffected (Suppl. Figure 2D-F). This illustrates the variability inherent in the RNAi method.

**Figure 5:**
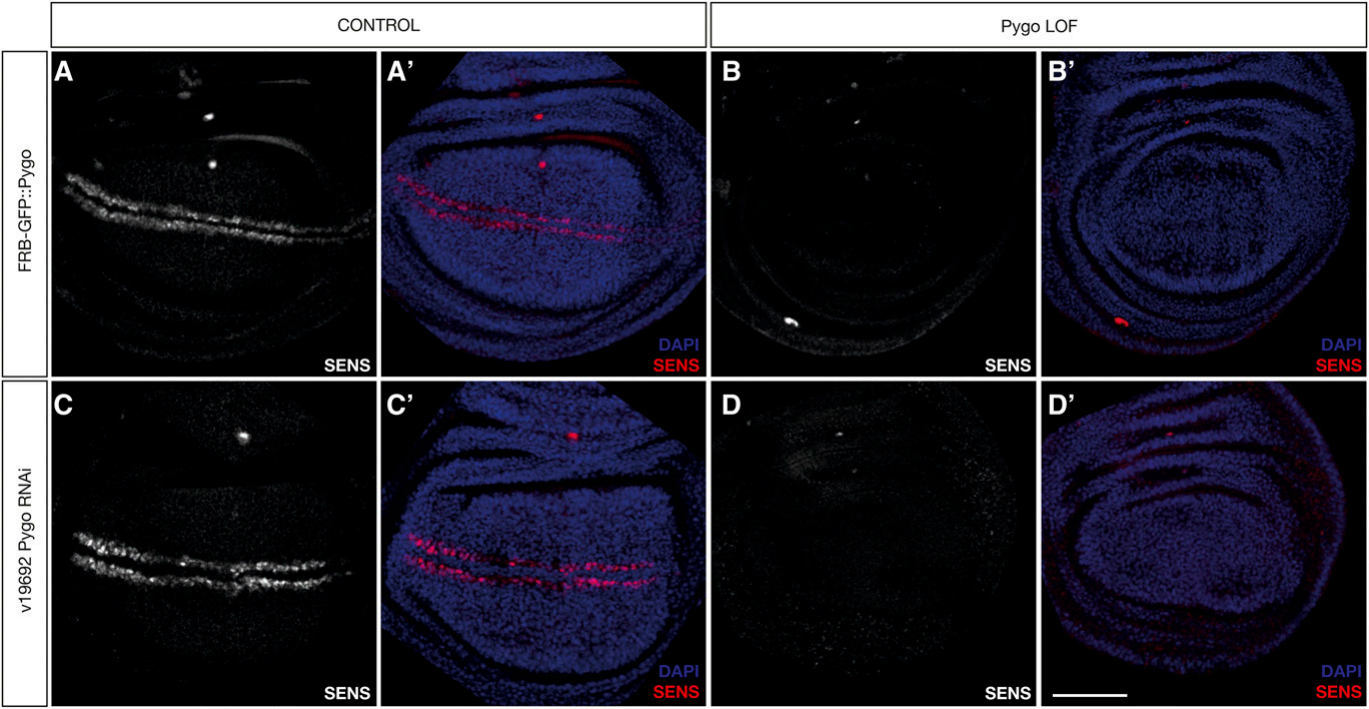
The Anchor-away yields LOF phenotypes as efficiently as RNAi. A-A’) FRBGFP::Pygo discs without rapamycin treatment. B-B’) FRBGFP::Pygo discs dissected 48 hr after rapamycin treatment. C-C’) Discs without induction of *pygo* RNAi. D-D’) Discs expressing *pygo* RNAi during 48 hr. Scalebar = 50 μM

The effects of the anchor-away system were more reliable, as Sens staining was never detected after rapamycin treatment in several independent tests of the system (Figure 5B-B’). Only for the situations in which RNAi worked, it was as effective as the anchor-away method (Figure 5D-D’, Suppl. Figure 2B). However, the variability exhibited by the different RNAi lines poses a real problem to perform LOF experiments.

All in all, our results show that the anchor-away is an efficient alternative method in *Drosophila* to perform LOF analyses.

## Discussion

The anchor-away technique has been developed in yeast (Haruki *et al.* 2008; Ding *et al.* 2014; Tsuchiya *et al.* 2013). In the current work, we successfully adapted the system to the model organism *Drosophila melanogaster*. The required transgenes were properly expressed, localized in their respective subcellular compartments, and preserved their molecular functions. We confirmed the efficiency of the technique to trap nuclear proteins. The strength of the LOF phenotypes indicates that complete inhibition was reached. The method also delivered a very fast effect, which was detectable within only a few hours (1 hr *ex vivo*, 6 hr *in vivo*).

RNAi knock-down will continue to be widely used for its simplicity and possibility to screen many genes. As a screening technique, it is thus unmatched thanks to the existing libraries (Dietzl *et al.* 2007; Ni *et al.* 2009) and simplicity of use, allowing for fast data acquisition and selection of candidate gene for further studies. However, when analyzing the function of a specific gene, the variability of the RNAi method has been a constant problem for researchers (DasGupta *et al.* 2007, Green *et al.* 2014). Off-target effects, or even dominant phenotypic effects, can affect the results of screens and LOF studies. The anchor-away method therefore represents a useful tool to rapidly induce a LOF in *Drosophila*.

Since its discovery, CRISPR/Cas9 has changed the time required to generate new alleles. It allows efficient generation of new transgenic strains or perform screens in a fraction of the time needed before (Hsu *et al.* 2014). Combined with CRISPR/Cas9, the anchor-away system can be adapted to any protein target in a time scale of weeks. CRISPR/Cas9 has also been used in *Drosophila* to perform genome-wide mutagenesis or overexpression screens (Ewen-Campen *et al.* 2017; Bassett *et al.* 2015). Following a similar approach, one could devise a genome-wide application of the anchor-away method, where potentially large libraries of FRB-tagged genes could be generated for screens in *Drosophila*.

By utilizing different anchors, other protein families could be sequestered from their subcellular compartments of residence. For example, cytoplasmic proteins could be sequestered to the plasma membrane (Tsuchiya *et al.* 2013). The anchor-away method could also be used to relocate proteins to different compartments and thereby force them to perform a secondary function, providing versatility to the technique. In summary, our adaption of the anchor-away system represents a useful addition to the toolbox of *Drosophila* researchers.
